# Obtaining Alumina from Kaolin Clay via Aluminum Chloride

**DOI:** 10.3390/ma12233938

**Published:** 2019-11-28

**Authors:** Vyacheslav I. Pak, Sergey S. Kirov, Anton Yu. Nalivaiko, Dmitriy Yu. Ozherelkov, Alexander A. Gromov

**Affiliations:** 1 Department of Non-ferrous Metals and Gold, National University of Science and Technology MISIS, Moscow 119991, Russia; pakvi@misis.ru (V.I.P.); kirovs13@yandex.ru (S.S.K.); 2 KINETICA Engineering Center, National University of Science and Technology MISIS, Moscow 119991, Russia; d.ozherelkov@gmail.com (D.Y.O.); a.gromov@misis.ru (A.A.G.)

**Keywords:** alumina, aluminum chloride hexahydrate, hydrochloric acid process, leaching, physico-chemical properties

## Abstract

A method of alumina production based on hydrochloric acid processing of kaolin clays from the East Siberian deposits was studied. Hydrochloric acid leaching was carried out at 160 °C. The leaching solution was subjected to a two-stage crystallization of aluminum chloride hexahydrate (ACH). The precipitated crystals were calcinated in air at a temperature above 800 °C to produce alumina. The main part of water and chlorine during thermal decomposition of ACH was removed at 400 °C. The influence of temperature and duration of ACH calcination on the residual chlorine content in alumina was studied. The optimal temperature of ACH calcination was 900 °C with a duration of 90 min. It was shown that the increase in calcination temperature contributed to the decrease in chlorine content in the final product. However, an increase in calcination temperature above 900 °C led to the transition of the well-soluble γ-Al_2_O_3_ phase to the insoluble α-Al_2_O_3_, which negatively affected the further electrolysis of aluminum. The size of alumina particles was not affected by the calcination mode. The rate of dissolution of the prototype Al_2_O_3_ in Na_3_AlF_6_ was higher than for the alumina obtained by the classical method. Alumina content, particle morphology, and particle size distribution for the obtained alumina were studied by X-ray diffraction (XRD), scanning electron microscopy (SEM), and laser diffraction methods. The obtained alumina is suitable for aluminum production according to the studied characteristics.

## 1. Introduction

Nowadays, the Bayer process is the predominant method of alumina production in the world. This method is based on the leaching of pre-crushed bauxite in alkaline solutions. After leaching, aluminum hydroxide separates from the aluminate solution and is then calcinated to produce alumina. High-quality bauxites with an Al_2_O_3_ to SiO_2_ mass ratio of more than 8 are required as a raw material for alumina production by the Bayer process [1]. The world’s reserves of high-quality bauxite reduce every year [2,3,4]. In this regard, a lot of intensive research focused on the expanding of the raw material base with the involvement of the low-quality aluminum raw materials (high-silica bauxite, kaolin clay, nepheline, ash) has been carried out [5,6,7,8].

The development of highly-efficient processing methods of low-quality aluminum raw materials with high silica content is an urgent task. One of the possible ways of high-silica raw materials processing is the sintering method. However, this method is multi-stage and characterized by high complexity as well as high fuel and reagents consumption. 

One of the most promising methods of obtaining alumina from high-silica aluminum ores is a hydrochloric acid technology, followed by the selective separation of aluminum compounds from acidic solutions [9,10,11]. The main advantage of this technology is the removal of silicon oxide at the first stage of the process. Silica separation helps to simplify the process and prevent the formation of harmful alumina production waste [12,13,14]. The advantage of hydrochloric acid as a leaching agent is the possibility of aluminum chloride hexahydrate (ACH) selective precipitation from leaching solutions. In addition, the hydrochloric acid method is the easiest in terms of acid regeneration compared to sulfuric and nitric acids. [15,16]. After the thermohydrolysis of aluminum chloride and iron crystals, gaseous HCl is captured by water and returned to leaching.

The purpose of this research is the study of the physical and chemical properties of alumina obtained by the hydrochloric acid technology using kaolin clays from Eastern Siberia, as well as the assessment of the obtained alumina suitability for aluminum electrolysis.

## 2. Materials and Methods 

### 2.1. Materials

A sample of kaolin clay from the East Siberian deposits was used as the raw material for alumina production. Table 1 shows the chemical composition of kaolin clay by the X-ray diffraction (XRD) method.

The initial samples of kaolin clay consisted of more than 50% silica, the content of aluminum oxide in the sample was 28.77%. Kaolin clay also contains oxides of iron, titanium, calcium, sodium, potassium, and magnesium. Experiments were carried out using hydrochloric acid (37 vol %). The required concentration of hydrochloric acid in the leaching process was achieved by diluting with distilled water. Hydrogen chloride gas was used in the crystallization process.

### 2.2. Characterization

Thermal analysis of the AlCl_3_·6H_2_O samples was performed using the STA 409 CD thermal analyzer (Netzsch Group, Selb, Germany) with a sample heating rate of 10 °C per minute in an argon gas atmosphere. The Vega LMH electron scanning microscope (Tescan, Brno, Czech Republic) with the Oxford Instruments Advanced AZtecEnergy analysis attachment (Asylum Research, NanoAnalysis, High Wycombe, UK) was used for the scanning electron microscopy (SEM) analysis of ACH crystals. Studies of the granulometric composition of alumina were carried out using the Microsizer-201S laser particle analyzer (Scientific instruments Jsc., Saint Petersburg, Russia). XRD of alumina was carried out using the D8 Advance equipment (Bruker Corp., Billerica, MA, USA). 

### 2.3. Experimental Research

**Figure 1:**
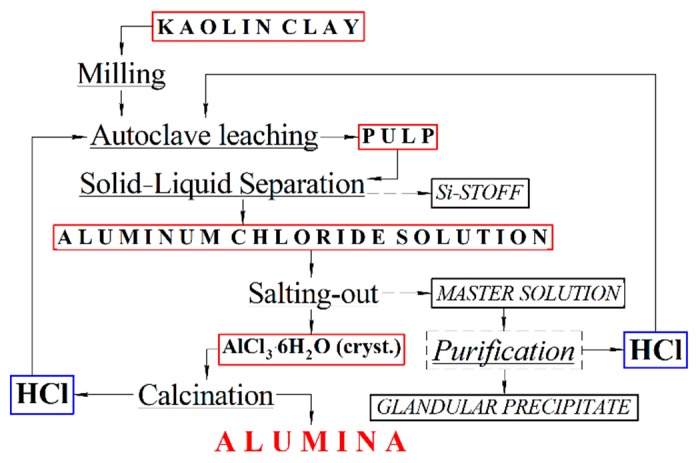
Technological scheme of alumina production from kaolin clays.

The technological scheme of the hydrochloric acid method for alumina production from kaolin clays is shown on Figure 1.

Leaching of kaolin clay was carried out for 3 h at 160 °C in an autoclave. Hydrochloric acid (20 vol %) was used as a leaching agent. The pulp after leaching was used for the thickening and filtering of Si-stoff. Si-stoff with a residual moisture content of 40–50 wt % was washed with water, which aimed to absorb HCl from the calcination gases. This process was followed by the obtainment of circulating acid. After washing, Si-stoff was sent for drying, and it can be used as a product for cement manufacturing.

After Si-stoff separation, the aluminum chloride solution was crystallized [17,18,19,20,21]. The process was carried out by saturating the aluminum chloride solution with gaseous HCl. Crystallization of AlCl_3_·6H_2_O was conducted in two stages. The first stage of crystallization was carried out at a temperature of 20 °C for 45 min in order to achieve a maximum degree of ACH crystallization (up to 97%). The crystals deposited at the first stage were recrystallized for 45 min to obtain crystals with low impurity content. AlCl_3_·6H_2_O crystals were calcinated in a tubular rotary furnace at a temperature of 900–1100 °C for 90 min [22,23].

**Figure 2:**
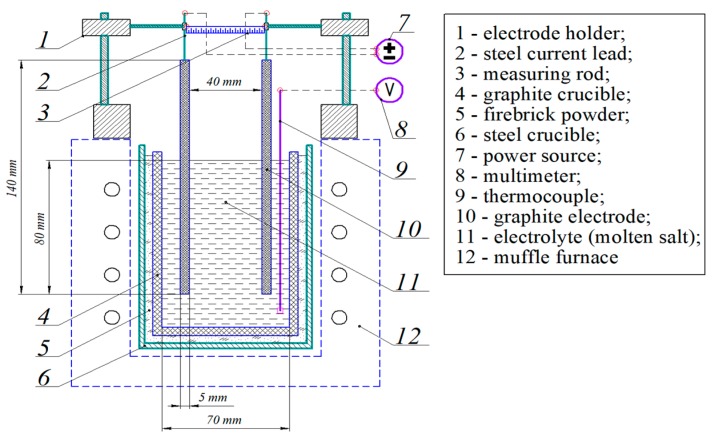
Scheme of the electrochemical cell.

An electrochemical cell was developed in order to determine the dissolution rate of Al_2_O_3_ in Na_3_AlF_6_. The scheme of this cell is shown in Figure 2. The graphite crucible was chosen as the basis for the electrochemical cell due to the stability in the aggressive molten cryolite salts [24,25,26]. The graphite crucible was enclosed in a steel casing and alumina backfill was located in the space between the crucible and casing. Graphite rods (0.5 cm × 14 cm) were used as electrodes. The measuring rod was installed in order to adjust the interelectrode distance between the electrode holders. The melt temperature was controlled by a platinum–platinum rhodium thermocouple. The GW PSW7 30-72 power source (GW instek, Taiwan) was used as a direct current (DC) power supply. The cell was heated using the KC 2/15 muffle furnace (Nabertherm GmbH, Lilienthal, Germany).

The electrodes were supplied with an electric current of a given density (I-const.). The change in the voltage on the electrochemical cell due to the addition of alumina to the melt was recorded until it reached a constant value. As an electrolyte composition close to the industrial electrolyte was used (5 wt % of Al_2_O_3_; 95 wt % of Na_3_AlF_6_ (2.7 cryolite ratio)), the melting point of this electrolyte was within the range of 945–960 °C [27]. To avoid melt freezing due to alumina addition (5 wt %), the experiment was carried out at an electrolyte temperature of 1100 °C.

The experiment was carried out at 0.5–1.0 A/cm^2^ current densities and at 4 cm of interelectrode distance.

## 3. Results and Discussion

The main reactions of kaolin clays during hydrochloric acid leaching were Equations (1) and (2):

Al_2_O_3_·2SiO_2_·2H_2_O + 6HCl → 2AlCl_3_ + 2SiO_2_↓ + 5H_2_O, (1)

Me_x_O_y_ + 2yHCl → xMeCl_(2y/x)_ + yH_2_O, (2)

where Me is Fe, Ca, K, Na, Mg.

As shown in Equations (1) and (2), aluminum dissolves in hydrochloric acid to form ACH. The amount of aluminum recovered into the hydrochloric acid solution was 95%. In this case, silica (the main component of kaolin clays) was not dissolved in hydrochloric acid and was separated from the solution by filtration. Other impurities also transferred into the leaching solution with a different extraction value. The composition of the aluminum chloride solution is presented in Table 2.

**Figure 3:**
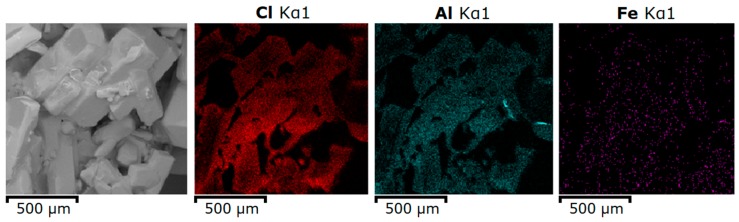
SEM images of aluminum chloride hexahydrate crystals.

After separation from the solid residue, the aluminum chloride solution was subjected to a two-stage crystallization of AlCl_3_·6H_2_O. Selective deposition of AlCl_3_·6H_2_O crystals occurred during crystallization and most of the metal chloride impurities remained in the solution. Precipitated crystals of AlCl_3_·6H_2_O were particles with a hexagonal form combined into agglomerates of different sizes (Figure 3) with a grain size of up to 550 microns.

**Figure 4:**
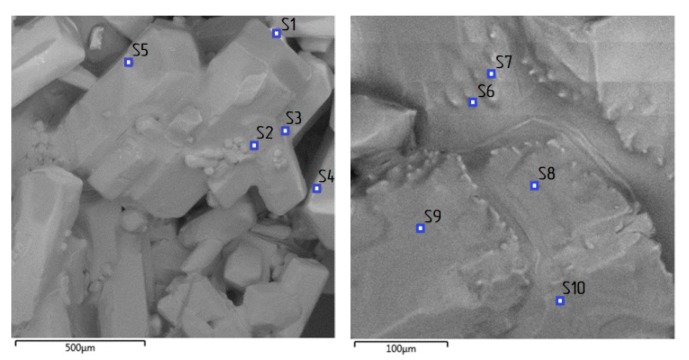
SEM images of aluminum chloride hexahydrate (ACH) with marked micro-XRD spectral analysis points.

The main impurity in the ACH crystals was iron. The main part of the iron was concentrated on the surface of the crystals and was represented by the residual master solution (see Figure 3). At the same time, part of the iron impurities was distributed point-wise throughout ACH crystals volume (see Figure 4 and Table 3).

**Figure 5:**
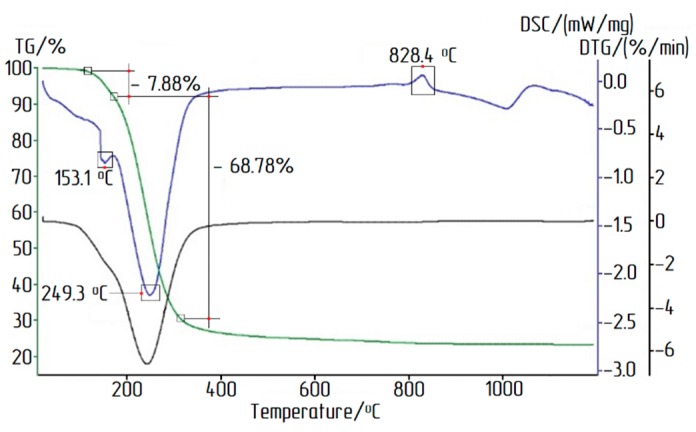
TG/DTA of AlCl_3_·6H_2_O sample in an argon atmosphere: TG mass loss curve, DTG mass loss rate curve, DSC differential scanning calorimetry curve.

**Figure 6:**
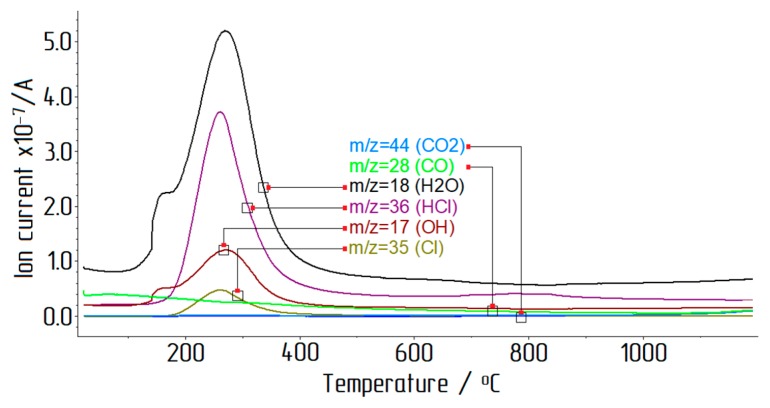
ACH synchronous thermal analysis: mass spectral ion current versus temperature.

To determine the temperatures of dehydration, water and chlorine removal, as well as phase transitions, the obtained samples of ACH were subjected to thermal analysis (see Figure 5 and Figure 6). The measurements were performed on the initial ACH under dynamic heating of the sample up to 1200 °C at a rate of 10 degrees per minute in an argon atmosphere.

The first stage of mass loss occurred between 105 °C and 156 °C and was associated with the removal of adsorbed water. Further heating of the sample (up to 1200 °С) removed water and chlorine from AlCl_3_·6H_2_O, with a mass loss of 76.66%. The main release of water and chlorine occurred up to 400 °C. At high temperatures, the DSC curve showed an exothermic peak associated with the beginning of γ-Al_2_O_3_ to α-Al_2_O_3_ transition. The thermal decomposition reaction of ACH crystals can be represented by the following Equation (3):

2AlCl_3_·6H_2_O → Al_2_O_3_ + 6HCl↑ + 9H_2_O↑. (3)

To determine the residual chlorine content in the products of ACH calcination, ACH thermal decomposition in a stationary mode at 450–1250 °C and a duration of 30–90 min was made. Calcination (in air) was carried out in the RT 50-250/13 tube furnace (Nabertherm GmbH, Lilienthal, Germany). The weight of the ACH sample was 10 g. The results of the study are presented in Table 4. The calcination temperature had the most influence on the residual chlorine content in rough alumina. The chlorine content decreased from 7.20% to 0.12% with increasing temperature from 450 °C to 1250 °C during the 30 min duration of the calcination process. With increasing duration from 30 to 90 min, the chlorine content reduced from 7.20% to 4.26% at the temperature of 450 °C. The minimum chlorine content in alumina (0.05 wt %) was obtained at 1250 °C and 90 min of calcination. The alumina obtained at 1250 °C was characterized by an increase in α-Al_2_O_3_ content, which adversely affected the subsequent electrolysis of aluminum. The calcination of ACH below 900 °C contributed to an increase of chlorine content in the final product.

According to the results of the thermal analysis of AlCl_3_·6H_2_O (see Figure 5), studies on the calcination of aluminum chloride hexahydrate crystals were carried out. The studies were conducted at three different calcination temperatures for 90 min each. The chemical compositions of alumina obtained by calcination are presented in Table 5.

**Figure 7:**
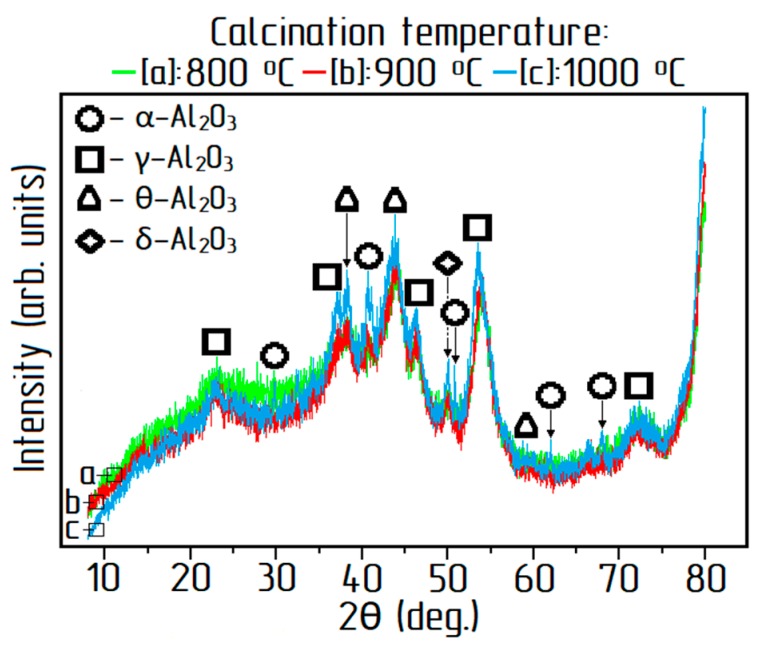
XRD patterns of alumina obtained at 800 °C, 900 °C, 1000 °C.

Various modifications of alumina were formed depending on the calcination temperature (see Figure 7). Alumina obtained at 800 °C is mainly represented by γ-Al_2_O_3_ with a small fraction of δ-Al_2_O_3_. Identical peaks were received at 900 °C. At 1000 °C there was a transition to θ-Al_2_O_3_ and α-Al_2_O_3_. These results were comparable to previous studies [28,29]. As can be seen in Table 5 and Figure 7, an increase in the calcination temperature above 900 °C led to a decrease in the chlorine content of the final product, but at the same time promoted the transition of γ-Al_2_O_3_ to α-Al_2_O_3_, which negatively affected the dissolution of the resulting alumina during electrolysis of aluminum.

To study the effect of temperature conditions on the particle size of alumina, experiments on calcination of ACH were carried out in two technological modes:

- With smooth heating of the material to the operating temperature for 2 h, followed by 2 h of exposure at operating temperature;
- Thermal shock-placing of the material into a hot furnace with a 4 hr exposure at operating temperature.

**Figure 8:**
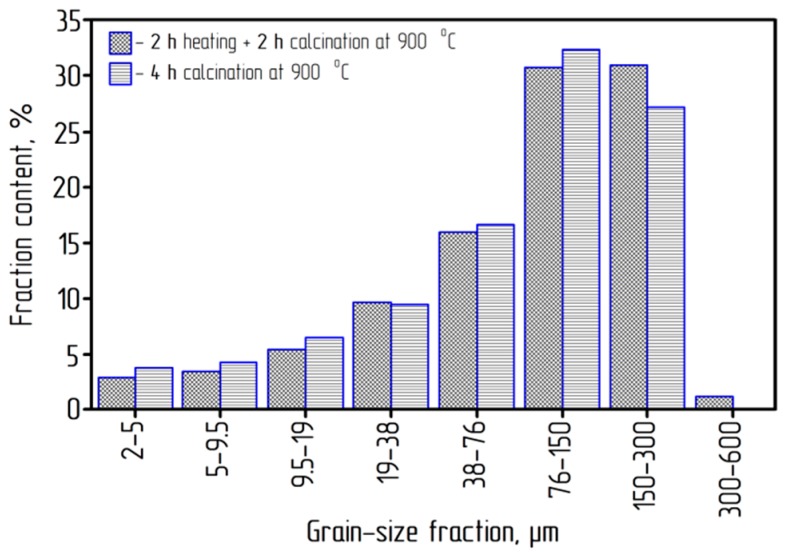
The size distribution of alumina (calcination process at 900 °C).

The operating temperature of the calcination was 900 °C. The ACH crystal size subjected to calcination was more than 450 microns. The size distribution of alumina obtained by calcination is shown in Figure 8.

The average size of alumina particles obtained at both temperature modes of calcination was 150 microns. Thus, the particle size decreased by more than three times during ACH calcination, which can be explained both by the destruction of agglomerates of the initial ACH and primary crystals. According to Figure 8, when the ACH was placed in a hot furnace, there was a slight over-grinding of the material compared to the smooth heating. This was due to the more intense release of the gas phase from the ACH.

**Figure 9:**
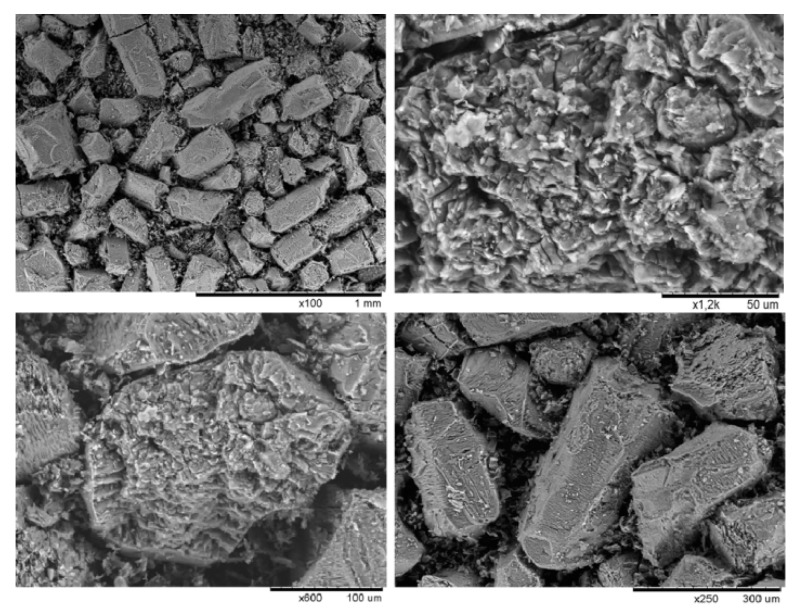
SEM images of alumina obtained by hydrochloric acid technology.

Figure 9 shows SEM images of alumina obtained by hydrochloric acid technology. The samples were obtained by the calcination of aluminum chloride hexahydrate at 900 °C. As can be seen on the figure, the surface of alumina obtained from ACH was covered by the net of thin cracks emerging from the grain body. These cracks promote the removal process of hydrogen chloride and water vapor. At the same time, crack development was observed in certain directions according to the structural anisotropy of the AlCl_3_·6H_2_O crystal, without significant destruction of the ACH particle’s original shape. The destruction of ACH particles during calcination occurred mainly due to the destruction of agglomerates consisting of a large number of fine fractions. In addition, the developed porous surface of alumina obtained from ACH had a high active surface and promoted adsorption of fluorine-containing waste gases of aluminum electrolysis and increased the rate of alumina dissolution in the electrolyte.

Table 6 shows the compliance of the impurity content in the test samples with the requirements for metallurgical alumina. The content of controlled impurities in test samples of alumina obtained by hydrochloric acid technology met the requirements, which demonstrated the possibility of aluminum electrolysis.

Alumina was produced according to the technological scheme (Figure 1) of kaolin clay processing by the hydrochloric acid technology. The following parameters were used to obtain alumina: leaching temperature was 160 °C with 3 h duration. Then, two-stage crystallization of ACH was carried out: at a temperature of 20 and 80 °C. The duration of both stages was 45 min. In accordance with the studies of ACH heat treatment, the optimal calcination mode was at 900 °C and 90 min duration. The alumina produced satisfied the following properties: Al_2_O_3_ content was 99.7 wt %, the content of impurities didn’t exceed maximum allowed values. Alumina was represented by γ-modification, the average particle size was 150 microns.

**Figure 10:**
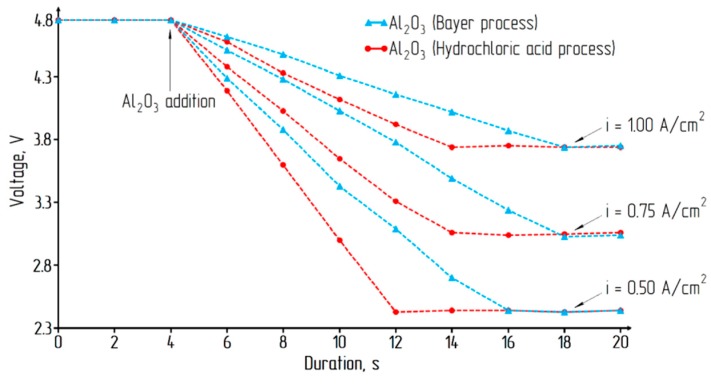
Voltage change in the electrochemical cell.

To assess the applicability of alumina obtained by the hydrochloric acid method, studies of its solubility in comparison with alumina produced by the classical Bayer process were made. The results of experimental studies are presented in Figure 10.

The achievement of a constant voltage on the electrochemical cell using alumina produced by acid technology occurred on average 4 s faster, which indicates a better solubility of the alumina in cryolite.

The electrochemical voltage consists of the voltage drop in the electrolyte (U_1_), the voltage drop in the contacts and conductors (U_2_), decomposition voltage (U_3_), and polarization voltage (U_4_) (4):

U = U_1_ + U_2_ + U_3_ + U_4_. (4)

Taking into account the small contact area of the current-carrying elements and small amperage, the voltage drop in the contacts and conductors (U_2_) was assumed to be zero. When alumina was added to the cryolite, the electrolysis reaction took the form (5):

Al_2_O_3_ + 1.5C → 2Al + 1.5CO_2_. (5)

Decomposition voltage (U_3_) in the temperature range of 1000–1100 °C was 1.15 V [30]. According to the results [31], the polarization voltage in the system С–СO_2_|Na_3_AlF_6_ (2.7–3.1 cryolite ratio) + Al_2_O_3_|Al-C at the current density of up to 1 A/cm^2^ is in the range of 0.03–0.06 V, with accepted 0.04 V.

The voltage drop in the electrolyte depends on the current density, conductivity of the electrolyte, and interelectrode distance, according to Formula (6):

U_1_ = ρ · i · l, (6)

where ρ is the resistivity of the electrolyte, Om·cm; i is the current density, A/cm^2^; and l is the interelectrode distance, cm.

The resistivity of the electrolyte at the points of voltage stabilization on the electrochemical cell was determined (Table 7).

According to Table 7, the resistivity of the used electrolyte, measured using the developed design of the electrochemical cell, was in the range of 0.623–0.637 Om·cm. This value was consistent with the literature data [32,33], which indicates the adequacy of the used method.

## 4. Conclusions

1. In this study, the residual chlorine content at calcination temperatures between 450 °C and 1250 °C with a duration between 30 min and 90 min was studied. The minimum chlorine content in alumina of 0.05 wt % was obtained at a temperature of 1250 °C and a calcination duration of 90 min, but the samples were characterized by an increase in the α-phase content, which adversely affected the subsequent electrolysis of aluminum. The calcination of ACH below 900 °C contributed to an increased chlorine content in the final product. The optimal mode of aluminum chloride hexahydrate calcination was determined as 90 min at 900 °C;
2. During thermal decomposition of ACH crystals, the main part of water and chlorine precipitated during the heating up to 400 °C;
3. The mode of calcination did not significantly affect the size of the obtained alumina. At the same time, the particle size during the calcination of ACH in both modes of calcination decreased by three times, from 450 microns for ACH to 150 microns for alumina;
4. The rate of Al_2_O_3_ in Na_3_AlF_6_ dissolution was studied. It was revealed that the dissolution of the alumina test sample in cryolite was faster than alumina obtained by the classical method (Bayer process).

## Figures and Tables

**Table 1:** The chemical composition of kaolin clay.

Component	SiO_2_	Al_2_O_3_	Fe_2_O_3_	TiO_2_	CaO	Na_2_O	K_2_O	MgO	Loss on Ignition
**Content, wt %**	50.71	28.77	6.14	0.42	0.43	0.18	0.23	0.72	12.40

**Table 2:** Chemical composition of the hydrochloric acid leaching solution.

Component	AlCl_3_	FeCl_3_	NaCl	KCl	CaCl_2_	MgCl_2_	HCl
**Content, wt %**	21.96	3.62	0.25	0.15	0.29	0.48	0.84

**Table 3:** Micro-XRD spectral analysis results for ACH crystals.

Spectrum Label	O	Al	Cl	Fe	Total
S1	50.66	31.89	17.03	0.42	100.00
S2	72.43	12.58	14.97	0.02	100.00
S3	69.69	11.22	19.03	0.06	100.00
S4	66.74	13.83	19.33	0.1	100.00
S5	55.06	16.69	28.20	0.05	100.00
S6	29.83	4.26	65.91	0.00	100.00
S7	57.84	12.70	29.37	0.09	100.00
S8	49.08	18.46	32.41	0.04	100.00
S9	53.38	17.00	29.58	0.04	100.00
S10	60.28	10.91	28.81	0.00	100.00

**Table 4:** The dependence of residual chlorine content from the parameters of the calcination.

Experiment Code	Temperature, ºС	Duration, min	Chlorine Content, wt %
1	450	30	7.20
2	450	60	6.00
3	450	90	4.26
4	900	30	1.70
5	900	60	0.91
6	900	90	0.22
7	1250	30	0.12
8	1250	60	0.08
9	1250	90	0.05

**Table 5:** Chemical composition of alumina obtained in different modes.

Temperature, °С	Al_2_O_3_	MgO	Na_2_O	K_2_O	CaO	SiO_2_	Fe_2_O_3_	Cl
800	99.362	0.043	0.003	0.010	0.028	0.005	0.015	0.534
900	99.678	0.047	0.003	0.007	0.029	0.007	0.020	0.209
1000	99.799	0.048	0.002	0.006	0.031	0.008	0.025	0.081

**Table 6:** Comparative evaluation of alumina impurities.

Component	Average Content, wt %	Industrial Requirements, wt %
SiO_2_	0.006	≤0.020
Fe_2_O_3_	0.020	≤0.030
Na_2_O	0.003	≤0.400 (Na_2_O + K_2_O)
K_2_O	0.007	
ТiO_2_	<0.001	≤0.010
CaO	0.029	Not specified
MgO	0.045	Not specified
Cl^−^	0.21	Not specified

**Table 7:** Calculation of the electrolyte resistivity.

Parameter	Current Density, A/cm^2^		
	0.50	0.75	1.00
Decomposition voltage, V	1.15	1.15	1.15
Polarization voltage, V	0.04	0.04	0.04
The voltage drop in the electrolyte, V	1.25	1.87	2.55
Total	2.44	3.06	3.74
Resistivity of the electrolyte, Om·cm	0.625	0.623	0.637
The parameters of electrolysis: interelectrode distance = 4 cm; temperature = 1200 °С; electrolyte composition = 5 wt % Al_2_O_3_; 95 wt % Na_3_AlF_6_ (2.7 cryolite ratio)			
